# Shortening Indwelling Catheterization After Vaginal Surgery for Pelvic Organ Prolapse: Results from a Prospective Randomized Trial

**DOI:** 10.3390/jcm14238295

**Published:** 2025-11-22

**Authors:** Tala Kordis, Ana Kofol, Mija Blaganje

**Affiliations:** 1 Division of Obstetrics and Gynaecology, Department of Gynaecology, University Medical Centre Ljubljana, 1000 Ljubljana, Slovenia; 2 Faculty of Medicine, University of Ljubljana, Vrazov trg 2, 1000 Ljubljana, Slovenia

**Keywords:** urinary retention, catheterization, urinary tract infection, pelvic organ prolapse surgery, anterior colporrhaphy

## Abstract

**Background/Objectives**: Pelvic organ prolapse (POP) is a common condition affecting women. When conservative treatment fails, surgical correction is indicated. Anterior colporrhaphy (AC) is a standard procedure for anterior vaginal wall prolapse repair. Postoperatively, an indwelling urinary catheter (IUC) is typically inserted to prevent urinary retention; however, prolonged catheterization is a known risk factor for urinary tract infection (UTI). This study aimed to evaluate whether postoperative catheterization can be safely shortened from 4 days to 24 h after vaginal POP surgery, and to compare the incidence of urinary retention and UTI between the two groups. **Methods**: A prospective randomized controlled trial was conducted, including 119 patients scheduled for AC for POP repair. All patients received an IUC after surgery and were randomized to catheter removal after either 24 h (group 1) or 4 days (group 2). Urinary retention was defined as a postvoid residual volume > 200 mL after IUC removal. UTI was diagnosed based on typical symptoms and a positive urine culture (≥10^5^ CFU/mL). **Results**: Data from 80 patients were analyzed. There were no statistically significant differences in catheter reinsertion rates (15% in group 1 vs. 7.5% in group 2, *p* = 0.288). The incidence of urinary retention was not influenced by the use of Kelly sutures, concomitant procedures, or patient age. No UTIs were confirmed in either group. Median hospital stay was significantly shorter in group 1 (3 [2–4] days vs. 4 [4–4] days, *p* < 0.001). **Conclusions**: Short-term catheterization following anterior colporrhaphy is not associated with increased risk of urinary retention or infection. Reducing catheterization duration results in a shorter hospital stay, which may lower healthcare costs and improve patient throughput.

## 1. Introduction

Pelvic organ prolapse (POP) is a common gynecological condition in women, most strongly associated with a history of vaginal delivery [1,2,3,4,5]. While many patients remain asymptomatic, others report sensations of vaginal bulging, pelvic heaviness, genital discomfort, dyspareunia, constipation, and urinary or fecal incontinence [2,6,7,8,9]. Initial management of POP typically involves conservative approaches, including lifestyle modification and pessary use, the latter being considered first-line therapy [1,2]. Surgical treatment is indicated when conservative measures fail or when patients have different expectations regarding symptom relief and anatomical correction [1,8,10]. Among available surgical techniques, anterior colporrhaphy (AC) remains one of the most commonly performed and least invasive transvaginal procedures for the correction of anterior vaginal wall prolapse (cystocele) [11]. AC may be performed as a standalone procedure or in combination with other vaginal reconstructive operations if multiple compartments are involved [12].

An indwelling urinary catheter (IUC) is typically inserted after AC to prevent postoperative urinary retention (POUR), which may result from transient bladder atony, postoperative edema, or excessive tissue tightening [13,14]. The placement of Kelly sutures around the bladder neck to prevent urinary incontinence can further obstruct the urethra and increase the risk of POUR [13,15,16]. Fear of pain during the first voiding can also contribute to pelvic floor muscle spasm and hinder successful voiding [17,18]. The reported incidence of POUR following gynecological surgery ranges between 2.5% and 43% [14], depending on surgical type, technique, anesthesia, postoperative analgesia, patient age, and degree of prolapse [14,19]. If untreated, urinary retention can lead to bladder overdistension and long-term voiding dysfunction. Ultrasound measurement of postvoid residual volume is considered the most reliable diagnostic method. There is no universally agreed cutoff method, but in practice, many gynecologic protocols use volumes >200 mL as a pragmatic threshold [14,20]. Although some ultrasound-based definitions may use a higher cutoff (300–500 mL) [21], we adapted a more conservative threshold of inadequate emptying in our protocol to ensure timely intervention and avoid prolonged catheterization in line with perioperative safety principles [10].

IUC is one of the most important risk factors for developing a complicated urinary tract infection (UTI). UTI is diagnosed when the patient with IUC has characteristic symptoms of infection and microbial growth ≥ 10 5 CFU/mL in urine sample. Symptoms of UTI include dysuria, a burning sensation while voiding, discharging frequent small amounts of urine throughout the day, a feeling of incomplete emptying of the bladder. According to the guidelines of the European Urological Association (EAU), UTI is caused by IUC if the patient presents with infection when the catheter is inserted or if the UTI occurs less than 48 h after the removal of the IUC [22]. The duration of the catheterization should be as short as possible [22,23]. The length of catheterization significantly affects the rate of bacteriuria; for each day the catheter is inserted the likelihood of infection increases by 3–7% [23].

At our institution, as in many others in the region, the standard practice has been to remove the IUC on the fourth postoperative day following AC [24,25,26,27,28]. Consequently, patients are typically hospitalized for four days after surgery. In the era of enhanced recovery and fast-track protocols, where shorter hospitalization is increasingly prioritized even after major operations, we sought to evaluate whether early catheter removal (after 24 h) is safe and feasible following anterior colporrhaphy [29,30].

## 2. Materials and Methods

This prospective, randomized, controlled study was approved by the National Medical Ethics Committee in August 2020. A total of 119 patients scheduled for POP surgery including AC were enrolled between May 2021 and April 2022 after providing written informed consent. A preliminary power analysis indicated that a sample size of 60 patients per group would provide at least 80% power (*p* = 0.05) to detect 20% difference in the incidence of POUR between groups, assuming a 10% POUR rate in the control group.

### 2.1. Study Design and Randomization

Patients were randomized into two groups using a computer-generated randomization table. Randomization was performed during the preoperative outpatient visit, which took place several weeks before the planned surgery. Each participant was assigned a randomization and consecutive identification number. Group 1 was assigned to catheter removal 24 h after surgery, while Group 2 underwent catheter removal on the fourth postoperative day.

### 2.2. Perioperative Management

Patients were admitted to the Gynecological department the evening prior to the planned surgery for routine laboratory testing. Intraoperative antibiotic prophylaxis consisted of either cefazolin 2 g or gentamicin 240 mg. Prolapse surgery involving AC was performed according to standard surgical principles [11,31], and an IUC was inserted at the end of the procedure.

In Group 1, the catheter was removed 24 h after surgery; in Group 2, removal was performed on 4th postoperative day. After catheter removal, patients were encouraged to void spontaneously. Six hours later, a transabdominal bladder scan (Bladder Scanner™, Verathon Inc., Bothell, WA, USA) was performed to assess residual urine volume. Urinary retention was defined as a postvoid residual volume greater than 200 mL, in which case the catheter was reinserted. A residual volume ≤ 200 mL was considered indicative of successful voiding, and no reinsertion was performed [2,10,23,32,33].

### 2.3. Microbiological Evaluation

During the first voiding after catheter removal, patients were instructed on how to collect a clean-catch midstream urine sample. Samples were sent for microbiological analysis. At the same time, patients were interviewed regarding the presence of urinary tract infection (UTI) symptoms, including dysuria, burning sensation during urination, frequent voiding of small volumes, and a sensation of incomplete bladder emptying.

UTI was confirmed when microbial growth ≥ 10^5^ CFU/mL was present in the urine sample accompanied by typical symptoms of infection [22]. Asymptomatic bacteriuria—defined as microbial growth ≥ 10^5^ CFU/mL without symptoms—was not treated, in accordance with established clinical recommendations [10,22,32].

### 2.4. Data Collection and Statistical Analysis

The primary study outcomes were the incidence of urinary retention and urinary tract infection. Secondary outcomes included: (1) the number of catheter reinsertions due to POUR; (2) the impact of concomitant prolapse procedures performed in addition to AC—such as posterior colporrhaphy, Manchester–Fothergill surgery, vaginal hysterectomy, laparoscopically assisted vaginal hysterectomy, colpopexy on sacrospinous ligament and TVT (tension free vaginal tape)—on the incidence of POUR; (3) use of Kelly sutures during AC and its association with POUR; (4) total duration of catheterization; (5) and length of hospital stay. The incidence of POUR per surgeon was also recorded.

Potential predictors of prolonged hospitalization were analyzed using multiple regression analysis. Continuous variables were compared using Student’s *t*-test or Mann–Whitney U test, and categorical variables using the chi-square test. A *p*-value < 0.05 was considered statistically significant. Statistical analysis was performed using SPSS software, version 27.0 (IBM Corporation, Armonk, NY, USA).

## 3. Results

**Figure 1:**
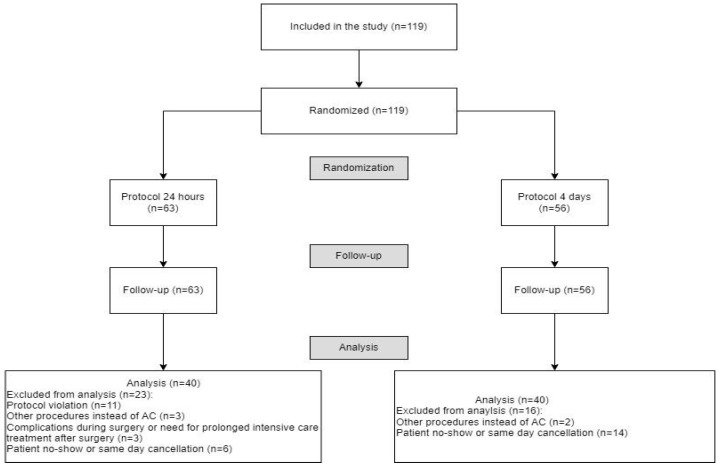
Flow-chart presenting patient enrollment, randomization, reasons for exclusion and the final number of patients in each group.

Between May 2021 and April 2022, 119 patients scheduled for AC for POP repair were enrolled in the study. In group 1, eleven protocol violations occurred in which the catheter was removed after more than 24 h; these patients were therefore excluded from the analysis. Patients were enrolled in the study several months before the planned surgery, and in some cases, the planned procedure was changed on the day of admission. If AC was not performed as scheduled, patients were excluded from the study. Three patients in group 1 experienced postoperative complications requiring prolonged intensive care treatment; in these cases, removal of the catheter was delayed until the patients were transferred from intensive care unit to the general ward. In both groups some procedures were cancelled when patients enrolled months earlier subsequently changed their minds and no longer wished to undergo surgery (Figure 1).

### 3.1. Patient Characteristics

Baseline characteristics were comparable between groups. There were no significant differences in age (mean ± SD: Group 1, 65.9 ± 9.9 years; Group 2, 64.5 ± 11.1 years; *p* = 0.552) or in the frequency of concomitant procedures performed together with AC (Table 1).

A total of 15 surgeons performed the operations, each operating on between one and twelve patients, with procedures evenly distributed among them.

### 3.2. Postoperative Urinary Retention

In the 24 h group, 6 of 40 patients (15.0%) required catheter reinsertion because of POUR, compared with 3 of 40 patients (7.5%) in the 4 days group (*p* = 0.288).

Kelly sutures were placed in 25 patients, 4 (16.0%) of whom required reinsertion of the IUC. Among the remaining 55 patients without Kelly sutures, 5 (9.1%) required reinsertion, with no significant difference between groups (*p* = 0.365).

Catheter reinsertion was necessary in 2 of 26 patients (7.7%) undergoing AC alone and in 7 of 54 patients (13.0%) who had concomitant procedures (*p* = 0.485). POUR incidence did not differ among surgeons, and no specific surgeon had a higher reinsertion rate.

The mean age of patients requiring reinsertion was 62.7 ± 7.0 years, compared to 65.5 ± 10.8 years among those who did not; higher age was not associated with an increased risk of POUR (*p* = 0.448).

### 3.3. Urinary Tract Infections

No UTIs were confirmed in either study group.

### 3.4. Duration of Catheterization and Hospital Stay

The duration of catheterization and hospitalization were both significantly shorter in the 24 h group (Table 2).

### 3.5. Hospitalization and Concomitant Procedures

Patients undergoing AC with additional procedures had a significantly longer hospital stay than those with isolated AC (Table 3 and Table 4).

In the 24 h group, mean hospital stay was 2.9 ± 0.9 days in patients without POUR and 3.8 ± 1.5 days in those requiring catheter reinsertion; the difference was not statistically significant (*p* = 0.203).

### 3.6. Predictions of Hospital Stay

Overall, hospital stay was significantly influenced by group allocation, presence of concomitant procedures, and occurrence of POUR. Patients in the 4 days group stayed on average 1.1 ± 0.2 days longer than those in the 24 h group (*p* < 0.001). If AC was performed as a stand-alone procedure, hospitalization was 0.6 ± 0.2 days shorter than when concomitant procedures were performed (*p* = 0.002). The presence of urinary retention prolonged hospitalization by 0.7 ± 0.3 days (*p* = 0.015).

## 4. Discussion

The results of our study indicate that there are no valid concerns regarding short-term catheterization following AC. No statistically significant differences were observed between the groups in terms of catheter reinsertion due to POUR or UTI rates. Furthermore, early catheter removal significantly reduced the duration of hospitalization.

Age of the patient, Kelly sutures, individual surgical technique and concomitant procedures besides AC are possible risk factors for developing urinary retention. Patients with POUR who required catheter reinsertion were not older than those who did not develop POUR (*p* = 0.448).

Four out of nine patients who developed POUR and needed catheter reinsertion had Kelly sutures, which was not statistically significant (*p* = 0.365), so it could not be confirmed Kelly sutures represented an independent risk factor for developing POUR.

Individual surgical techniques could have an impact on the outcome of the surgery [19]. In our study, there were no significant differences in incidence of POUR per surgeon.

Seven of the nine patients (78%) who required catheter reinsertion due to POUR underwent concomitant procedures such as vaginal hysterectomy with salpingectomy, posterior colporrhaphy, tension-free vaginal tape (TVT), Manchester–Fothergill, or sacrospinous ligament colposuspension. Among patients who underwent AC alone, 2 of 26 (7.7%) developed urinary retention, compared with 7 of 54 (13.0%) in the concomitant-surgery group. This difference was not statistically significant (*p* = 0.485), indicating that additional surgical procedures did not substantially increase the risk of POUR.

Six of the nine patients who developed POUR required catheter reinsertion more than once before successful voiding. All required catheterization for longer than four days (ranging from 9 to 21 days), suggesting that these patients likely would have developed POUR regardless of their initial group assignment.

A major strength of our study is the use of European Association of Urology (EAU) criteria for defining UTI, unlike other studies that considered positive urine culture alone as diagnostic [13,17,34,35]. Since only symptomatic UTIs warrant treatment, using the EAU definition helps avoid unnecessary antibiotic prescriptions, thereby improving patient safety and reducing the risk of antimicrobial resistance [36]. Although our study found no significant difference in UTI rates between the 24 h and 4 days catheterization protocols, given the established theoretical risk of bacteriuria increasing by 3–7% for each day of catheterization, early catheter removal appears clearly advantageous [23].

Some bias may have occurred due to missing urine samples. Routine urine sampling after catheter removal is not standard practice in our institution, and compliance with the protocol varied between groups. Nurses were more diligent in collecting samples from the 24 h group, as this was a new protocol requiring greater attention, whereas collection in the 4 days group, which reflected standard practice, was less consistent. Consequently, urine samples were obtained in 70% of group 1 patients and 42.5% of group 2. Nevertheless, none of the patients exhibited clinical symptoms of UTI, supporting the conclusion that no infections were present.

The duration of catheterization was, by design, significantly shorter in group 1 (*p* < 0.001), which led to a correspondingly shorter hospital stay. Mean hospitalization in the 24 h group was one day shorter than in the 4 days group (*p* < 0.001). Concomitant procedures increased hospital stay in both groups. In the 24 h group, patients who underwent AC alone were hospitalized for an average of 2.4 days, compared to 3.4 days for those with additional procedures (*p* < 0.001). The occurrence of urinary retention did not significantly prolong hospitalization in group 1.

Shorter hospital stays can substantially reduce treatment costs. In an era of minimally invasive and fast-track surgery, optimizing cost-efficiency without compromising quality of care is a shared priority. Reduced length of stay can enhance patient satisfaction while improving institutional efficiency [37,38,39,40,41].

A limitation of our study is smaller than planned sample size (80 patients included in the final analysis), resulting in reduced power. Between March 2020 and May 2022, elective surgical programs in our institution were reduced by approximately 50% due to the COVID-19 pandemic. National and international recommendations prioritized only urgent surgeries during this period [42,43,44]. Global predictive models estimated that during the first 12 weeks of lockdown, approximately two million operations were cancelled or postponed worldwide each week [44]. The pandemic also increased preoperative anxiety and patient cancellations due to fear of infection or active COVID-19 illness, further reducing the recruitment rate for elective studies such as ours [45,46,47,48,49]. The observed difference between groups for POUR should therefore be interpreted with caution.

Our findings support the implementation of early catheter removal—within 24 h—after anterior colporrhaphy as a safe and effective approach that aligns with the principles of enhanced recovery after surgery (ERAS). Short-term catheterization does not increase the risk of urinary retention or infection, while significantly reducing hospitalization time. ERAS protocols recommend removal of IUC as early as safely possible, often within the first 24 h on first postoperative day [50]. We acknowledge that perioperative practices differ between institutions. Our practice and ERAS protocol recommendations can serve as a model for other centers: institutions currently using longer-duration catheterization, may consider reviewing and shortening their protocol towards 24 h or less, provided their institutional infrastructure allows safe implementation. Incorporating this protocol into standard postoperative care could streamline patient management, improve comfort and satisfaction, and contribute to resource optimization in urogynecologic practice.

## 5. Conclusions

No significant disadvantages were identified with short-term catheterization following anterior colporrhaphy with or without concomitant POP surgery. There were no statistically significant differences in the incidence of urinary retention requiring catheter reinsertion or in UTI rates between the 24 h and 4 days catheterization groups. Implementing a shorter catheterization protocol after AC can safely reduce hospital stay, decrease treatment costs, and improve patient throughput without compromising surgical outcomes.

## Figures and Tables

**Table 1:** Types of performed surgeries.

Type of Surgery	24 h Group (n = 40)	4 Days Group (n = 40)
Only anterior colporrhaphy	13	13
Anterior and posterior colporrhaphy	3	4
Anterior colporrhaphy and Manchester–Fothergill surgery	5	0
Anterior colporrhaphy and vaginal hysterectomy	9	18
Anterior colporrhaphy and LAVHA	3	1
Anterior colporrhaphy and colpopexy on sacrospinous ligament	3	0
Anterior colporrhaphy and TVT	4	4

Data are presented as the number of patients. LAVHA: laparoscopically assisted vaginal hysterectomy with adnexectomy, TVT: tension free vaginal tape.

**Table 2:** Duration of catheterization and hospitalization.

	24 h (n = 40)	4 Days (n = 40)	*p*-Value
Duration of catheterization (days)	1 [1–1]	4 [4–4]	<0.001
Duration of hospitalization (days)	3 [2–4]	4 [4–4]	<0.001

Data are presented as median (IQR).

**Table 3:** Duration of hospitalization in patients with AC and concomitant procedures.

	Concomitant Procedures (n = 54)	AC (n = 26)	*p*-Value
All patients (days)	3.8 (0.9)	3.1 (1.1)	0.010
24 h group (days)	3.4 (1.0)	2.4 (0.8)	0.001
4 days group (days)	4.2 (0.6)	3.7 (0.9)	0.339

Data are presented as average (SD). AC-anterior colporrhaphy.

**Table 4:** Duration of hospitalization in patients with AC only by catheterization group.

	24 h, AC (n = 13)	4 Days, AC (n = 13)	*p*-Value
Duration of hospitalization (days)	2.4 (0.8)	3.9 (0.9)	<0.001

Data are depicted as average (SD). AC-anterior colporrhaphy.

## Data Availability

The data presented in this study are available on request from the corresponding author. The data are not publicly available due to patient privacy.

## Abbreviations

The following abbreviations are used in this manuscript:

POP	Pelvic organ prolapse
AC	Anterior colporrhaphy
IUC	Indwelling urinary catheter
POUR	Postoperative urinary retention
UTI	Urinary tract infection
TVT	Tension-free vaginal tape
EAU	European Association of Urology
ERAS	Enhanced recovery after surgery

## References

[1] Iglesia C.B., Smithling K.R. (2017). Pelvic Organ Prolapse. Am. Fam. Physician.

[2] Dietz H.P. (2015). Pelvic organ prolapse—A review. Aust. Fam. Physician.

[3] Jelovsek J.E., Maher C., Barber M.D. (2007). Pelvic organ prolapse. Lancet.

[4] Urbankova I., Grohregin K., Hanacek J., Krcmar M., Feyereisl J., Deprest J., Krofta L. (2019). The effect of the first vaginal birth on pelvic floor anatomy and dysfunction. Int. Urogynecol. J..

[5] Blomquist J.L., Muñoz A., Carroll M., Handa V.L. (2018). Association of Delivery Mode With Pelvic Floor Disorders After Child-birth. JAMA.

[6] Weintraub A.Y., Glinter H., Marcus-Braun N. (2020). Narrative review of the epidemiology, diagnosis and pathophysiology of pelvic organ prolapse. Int. Braz. J. Urol..

[7] Serdinšek T., But I. (2019). Začetna obravnava bolnice z uroginekološkimi težavami. Slov. Med. J..

[8] American College of Obstetricians and Gynecologists, The American Urogynecologic Society (2019). Pelvic Organ Prolapse. Female Pelvic Med. Reconstr. Surg..

[9] Barber M.D., Maher C. (2013). Epidemiology and outcome assessment of pelvic organ prolapse. Int. Urogynecol. J..

[10] Haya N., Feiner B., Baessler K., Christmann-Schmid C., Maher C. (2018). Perioperative interventions in pelvic organ prolapse sur-gery. Cochrane Database Syst. Rev..

[11] Altman D., Väyrynen T., Engh M.E., Axelsen S., Falconer C. (2011). Anterior colporrhaphy versus transvaginal mesh for pelvic-organ prolapse. N. Engl. J. Med..

[12] Loh K.C., Umanskiy K. (2021). Ventral Rectopexy. Clin. Colon Rectal Surg..

[13] Weemhoff M., Wassen M.M.L.H., Korsten L., Serroyen J., Kampschöer P.H.N.M., Roumen F.J.M.E. (2011). Postoperative catheterization after anterior colporrhaphy: 2 versus 5 days. A multicentre randomized controlled trial. Int. Urogynecol. J..

[14] Geller E.J. (2014). Prevention and management of postoperative urinary retention after urogynecologic surgery. Int. J. Womens Health.

[15] Hakvoort R.A., Dijkgraaf M.G., Burger M.P., Emanuel M.H., Roovers J.P.W.R. (2009). Predicting Short-Term Urinary Retention After Vaginal Prolapse Surgery. Neurourol. Urodyn..

[16] Trowbridge E.R., Buchanan L.E., Evans S.L., Allen M.N., Chacon H.L., Hullfish K.L. (2022). Pass or Fail? Postoperative Active Void-ing Trials in an Enhanced Recovery Program. Female Pelvic Med. Reconstr. Surg..

[17] Hakvoort R.A., Elberink R., Vollebregt A., Ploeg T., Emanuel M.H. (2004). How long should urinary bladder catheterisation be continued after vaginal prolapse surgery? A randomised controlled trial comparing short term versus long term cathe-terisation after vaginal prolapse surgery. BJOG.

[18] Foster R.T., Borawski K.M., South M.M., Weidner A.C., Webster G.D., Amundsen C.L. (2007). A randomized, controlled trial evalu-ating 2 techniques of postoperative bladder testing after transvaginal surgery. Am. J. Obstet. Gynecol..

[19] Moen M., Noone M., Vassallo B. (2014). Anterior colporrhaphy: Why surgeon performance is paramount. Int. Urogynecol. J..

[20] Baldini G., Bagry H., Aprikian A., Carli F. (2009). Postoperative urinary retention: Anesthetic and perioperative considerations. Anesthesiology.

[21] Ceratti R.D.N., Beghetto M.G. (2021). Incidence of urinary retention and relations between patient’s complaint, physical exami-nation, and bladder ultrasound. Rev. Gaucha. Enferm..

[22] European Association of Urology (2022). EAU Guidelines on Urological Infections.

[23] Flores-Mireles A., Hreha T.N., Hunstad D.A. (2019). Pathophysiology, Treatment, and Prevention of Catheter-Associated Urinary Tract Infection. Top. Spinal Cord Inj. Rehabil..

[24] Huang C.C., Ou C.S., Yeh G.P., der Tsai H., Sun M.J. (2011). Optimal duration of urinary catheterization after anterior colporrhaphy. Int. Urogynecol. J..

[25] Kringel U., Reimer T., Tomczak S., Green S., Kundt G., Gerber B. (2010). Postoperative infections due to bladder catheters after an-terior colporrhaphy: A prospective, randomized three-arm study. Int. Urogynecol. J..

[26] Halpern-Elenskaia K., Umek W., Bodner-Adler B., Hanzal E. (2018). Anterior colporrhaphy: A standard operation? Systematic review of the technical aspects of a common procedure in randomized controlled trials. Int. Urogynecol. J..

[27] Lensen E.J.M., Stoutjesdijk J.A., Withagen M.I.J., Kluivers K.B., Vierhout M.E. (2011). Technique of anterior colporrhaphy: A Dutch evaluation. Int. Urogynecol. J..

[28] Fernandez-Gonzalez S., Martinez Franco E., Martínez-Cumplido R., Molinet Coll C., Ojeda González F., Gómez Roig M.D., Tardiu L.A. (2019). Reducing postoperative catheterisation after anterior colporrhaphy from 48 to 24 h: A randomised controlled trial. Int. Urogynecol. J..

[29] Blaganje M., Lutfallah F., Deval B. (2016). Mini-laparoscopic sacrocolpopexy for apical and posterior female pelvic organ pro-lapse. Int. Urogynecol. J..

[30] Hickman L.C., Paraiso M.F.R., Goldman H.B., Propst K., Ferrando C.A. (2021). Same-Day Discharge After Minimally Invasive Sac-rocolpopexy Is Feasible, Safe, and Associated With High Patient Satisfaction. Female Pelvic Med. Reconstr. Surg..

[31] Haylen B.T., Maher C.F., Barber M.D., Camargo S., Dandolu V., Digesu A., Goldman H.B., Huser M., Milani A.L., Moran P.A. (2016). An International Urogynecological Associa-tion (IUGA)/International Continence Society (ICS) Joint Report on the Terminology for Female Pelvic Organ Prolapse (POP). Neurourol. Urodyn..

[32] Geerlings S.E. (2016). Clinical Presentations and Epidemiology of Urinary Tract Infections. Microbiol. Spectr..

[33] Huang H., Dong L., Gu L. (2020). The timing of urinary catheter removal after gynecologic surgery: A meta-analysis of random-ized controlled trials. Medicine.

[34] Sekhavat L., Farajkhoda T., Davar R. (2008). The effect of early removal of indwelling urinary catheter on postoperative urinary complications in anterior colporrhaphy surgery. Aust. New Zealand J. Obstet. Gynaecol..

[35] Castillo-Pino E., Benavides N., Acevedo V., Alonso V. (2021). Removal time of postoperative vesical catheter in utero-vaginal prolapse surgery: A comparative study. Pelviperineology.

[36] Habboush Y., Guzman N. (2022). Antibiotic Resistance.

[37] Scheib S.A., Thomassee M., Kenner J.L. (2019). Enhanced Recovery after Surgery in Gynecology: A Review of the Literature. J. Minim. Invasive Gynecol..

[38] Ottesen M., Sørensen M., Rasmussen Y., Smidt-Jensen S., Kehlet H., Ottesen B. (2002). Fast track vaginal surgery. Acta. Obstet. Gynecol. Scand..

[39] Lambat Emery S., Brossard P., Petignat P., Boulvain M., Pluchino N., Dällenbach P., Wenger J.-M., Savoldelli G.L., Rehberg-Klug B., Dubuisson J. (2021). Fast-Track in Minimally Invasive Gynecology: A Randomized Trial Comparing Costs and Clinical Outcomes. Front. Surg..

[40] Nezhat C., Main J., Paka C., Soliemannjad R., Parsa M.A. (2014). Advanced gynecologic laparoscopy in a fast-track ambulatory surgery center. JSLS.

[41] Wodlin N.B., Nilsson L. (2013). The development of fast-track principles in gynecological surgery. Acta Obstet. Gynecol. Scand..

[42] COVIDSurg Collaborative (2020). Global guidance for surgical care during the COVID-19 pandemic. Br. J. Surg..

[43] Fowler A.J., Dobbs T.D., Wan Y.I., Laloo R., Hui S., Nepogodiev D., Bhangu A., Whitaker I.S., Pearse R.M., Abbott T.E.F. (2021). Resource requirements for reintroducing elective surgery during the COVID-19 pandemic: Modelling study. Br. J. Surg..

[44] Nepogodiev D., Bhangu A., COVIDSurg Collaborative (2020). Elective surgery cancellations due to the COVID-19 pandemic: Global predictive modelling to inform surgical recovery plans. Br. J. Surg..

[45] Chan J.J., Chen K.K., Choi P., Rojas E.O., Schipper O.N., Aiyer A., Netto C.d.C., Haleem A.M., Kadakia A.R., Vulcano E. (2021). Impact of COVID-19 Pandemic on Patients’ Perceptions of Safety and Need for Elective Foot and Ankle Surgery in the United States. Foot Ankle Orthop..

[46] Norris Z.A., Sissman E., O’Connell B.K., Mottole N.A., Patel H., Balouch E., Ashayeri K., Maglaras C., Protopsaltis T.S., Buckland A.J. (2021). COVID-19 pandemic and elective spinal surgery cancelations—What happens to the patients?. Spine J..

[47] Chang J., Wignadasan W., Kontoghiorghe C., Kayani B., Singh S., Plastow R., Magan A., Haddad F. (2020). Restarting elective orthopaedic services during the COVID-19 pandemic. Bone Jt. Open.

[48] Madanipour S., Al-Obaedi O., Ayub A., Iranpour F., Subramanian P. (2021). Resuming elective hip and knee arthroplasty in the COVID-19 era: A unique insight into patient risk aversion and sentiment. Ann. R. Coll. Surg. Engl..

[49] Garde-García H., González-López R., González-Enguita C. (2020). Functional urology surgery and SARS-CoV-2: How and why surgical activity should be resumed now, adapting to the new reality. Actas Urol. Esp..

[50] Silva Filho A.L.D., Santiago A.E., Derchain S.F.M., Carvalho J.P. (2018). Enhanced Recovery After Surgery (ERAS): New Concepts in the Perioperative Management of Gynecologic Surgery. Rev. Bras. Ginecol. Obstet..

